# Protracted circum-continent subduction: A mechanism for craton destruction and a rationale for craton longevity

**DOI:** 10.1073/pnas.2502618122

**Published:** 2025-08-07

**Authors:** Xi Xu, Andrew V. Zuza, Lijun Liu, Weilin Zhu, Yanyun Sun, Baodi Wang, Xingtao Kuang, Song Han, Xuanjie Zhang, Wan Zhang, Xiaowei Fu, D. Graham Pearson, Jingao Liu

**Affiliations:** ^a^College of Geophysics and Petroleum Resources, and Key Laboratory of Exploration Technologies for Oil and Gas Resources (Ministry of Education), Yangtze University, Wuhan 430100, China; ^b^China Aero Geophysical Survey and Remote Center for Natural Resources, China Geological Survey, Beijing 100083, China; ^c^School of Ocean and Earth Science, Tongji University, Shanghai 200092, China; ^d^Nevada Bureau of Mines and Geology, Nevada Geosciences, University of Nevada, Reno, NV 89557; ^e^State Key Laboratory of Lithospheric Evolution, Institute of Geology and Geophysics, Chinese Academy of Science, Beijing 100191, China; ^f^China National Offshore Oil Corporation, Beijing 100010, China; ^g^Department of Earth and Atmospheric Science, University of Alberta, Edmonton, AB T6G 2E3, Canada; ^h^State Key Laboratory of Geological Processes and Mineral Resources, and Frontiers Science Center for Deep-time Digital Earth, China University of Geoscience (Beijing), Beijing 100083, China

**Keywords:** craton, craton destruction, plate subduction, aeromagnetic imaging, lithosphere evolution

## Abstract

The evolution of continents is shaped by the growth and destruction of long-lived cratons, which serve as their stable cores. Processes for craton destruction are controversial because most invoked mechanisms occur frequently throughout Earth history, making the preservation of cratons for billions of years problematic. Here, we address this issue by presenting a crustal-scale analytical signal-amplitude model obtained from high-resolution airborne and shipborne magnetic data across cratons within East Asia. Magmatic, magnetic, and basin-history constraints show that the eastern North China craton experienced focused weakening, thickening, and catastrophic destruction of its mantle lithosphere due to a unique combination of circum-craton subduction and subsequent collision since the Paleozoic. By contrast, the adjacent South China craton was not impacted in this way, and thus, its mantle root was spared from destruction. The long-term survival of cratons may stem from the infrequent occurrence of sustained circum-cratonic subduction or collisional processes capable of destabilizing their lithospheric roots.

## Significance

The longevity of cratons is attributed to their refractory, buoyant mantle roots. Several cratons are argued to have recently lost their cratonic roots due to oceanic subduction, which is at odds with the observed survival of cratons for billions of years. We examined aeromagnetic surveys, basin histories, and Mesozoic magmatism to document spatially variable lithospheric modification across East Asia during Pacific subduction. The South China craton has been partially modified, whereas the eastern North China craton is completely destroyed. We attribute these differences to North China experiencing protracted >500 My circum-craton subduction. This suggests that craton destruction was a unique process of tectonic attrition, requiring unusual circumstances and offering a rationale for why most cratons persist throughout Earth history.

**Fig. 1.**
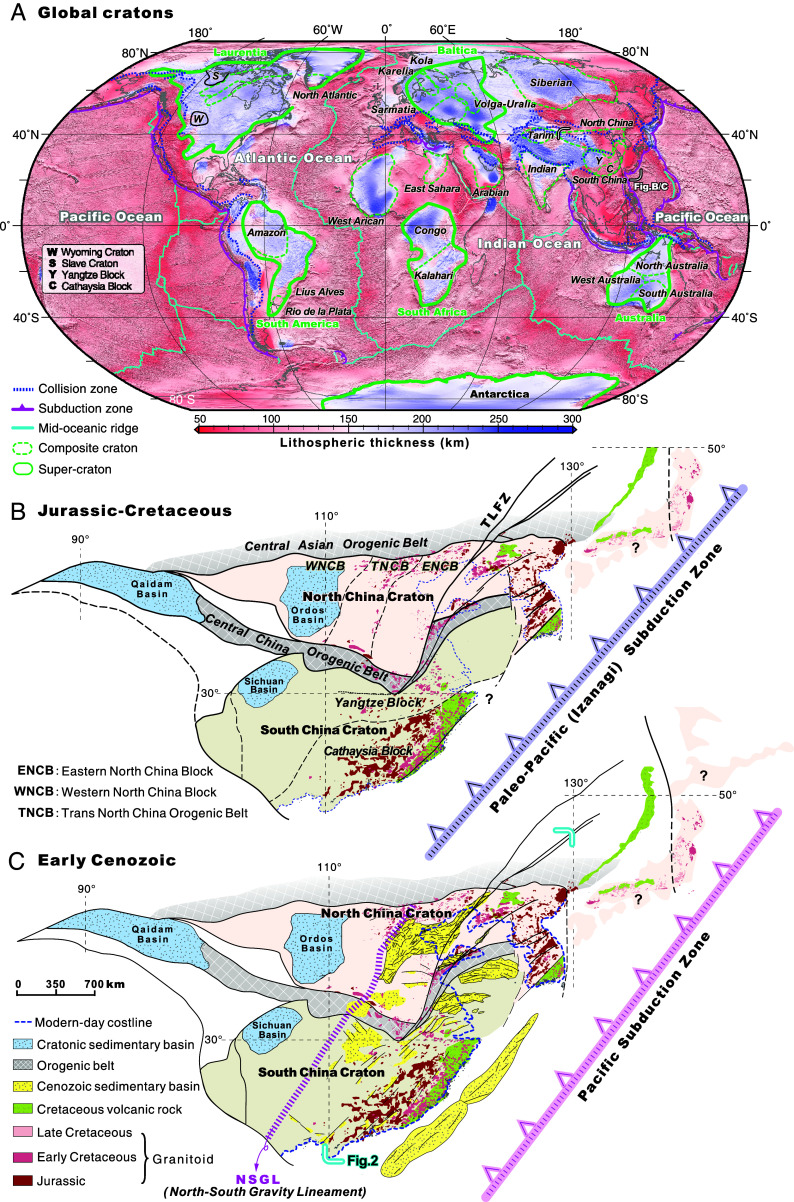
(*A*) Global cratonic regions with various lithospheric thickness. High-resolution global lithospheric thickness map produced using the SL2013sv tomography model (modified from 16). Lithospheric boundaries of cratons, including composite cratons and supercratons, are derived from (1). Tectonic boundaries, including collision and subduction zones and mid-oceanic ridges, are plotted from a global tectonic model (17). Tectonic framework of the North China and South China cratons (SCCs) (*B*) during the Jurassic-Cretaceous and (*C*) during the Early Cenozoic (7, 18, 19). Location of the reaserch area (Fig. 2) is also shown. Modern-day coastline is marked with blue dotted line.

Despite comprising over 60% of the continental landmass as the oldest nuclei of the Earth’s continents, the evolution of cratons remains hotly debated (Fig. 1*A*) (1). Cratonic mantle roots are compositionally depleted, buoyant, thick (>200 km), cold, and mechanically strong characteristics that enable the cratonic crust and lithospheric roots to remain largely intact for billions of years (2–4) while being repeatedly involved in the amalgamation and breakup of Earth’s major supercontinents (5). Despite this longevity, it is postulated that some cratons, such as the North China, Wyoming, and Amazonian cratons, have recently lost much of their ancient mantle roots, with their crustal sections extensively intruded by Phanerozoic magmatic products (Fig. 1*A*) (1, 6, 7). Deciphering potential mechanisms for craton modification is a critical requirement for understanding the long-term survival and evolution of continental mantle throughout Earth history. Craton destruction may progress via thermomechanical erosion by a hot mantle plume (8, 9) or through subduction zone weakening processes (6, 7, 10). Conversely, the modified lithospheric mantle of a craton may be healed via mantle plume processes (11–13) or subduction-associated relamination (14, 15). Mantle-plume activity and subduction processes have been persistent geodynamic processes throughout much of Earth’s history. Hence, if these mechanisms are effective at disrupting cratons, craton modification and destruction should be a relatively common event. This inference is clearly at odds with observations of the long-term stability of cratons since the Archean-Paleoproterozoic (1).

The Mesozoic destruction of the North China craton (NCC) is thought to represent an archetypical example of subduction-based craton destruction (6, 7, 10). Two end-member models have been proposed for the destruction and thinning of the NCC lithosphere (20, 21). First, subduction of the (Paleo-) Pacific oceanic plate may have driven upwelling of the asthenospheric mantle to cause bottom–up thermal–chemical erosion and removal of the cratonic mantle root (22, 23). Alternatively, the cratonic lithosphere may have been delaminated following eclogitization of the lower crust and underlying portions of the continental lithosphere, possibly triggered or accelerated by subduction processes (20, 24, 25). However, many unanswered questions and contradictory interpretations remain regarding the NCC destruction. Seismic imaging and mantle xenolith studies reveal that not all of the cratonic mantle along the eastern margin of Asia that borders the (Paleo-) Pacific subduction zone has been destroyed (6, 7), including major regions across the South China craton (SCC) to the south-southeast of the NCC (14, 26). If Paleo-Pacific subduction process was the sole force involved in destroying the mantle root of the NCC, this process should have similarly modified the adjacent SCC, located along-strike within the same subduction system. Understanding how certain cratons or craton portions remain unaffected by lithospheric modification provides critical insights into the overall process of craton evolution.

In the last few decades, observations from onshore seismic tomography and mantle xenoliths have offered significant insights into the temporal evolution of the NCC and SCC cratonic lithosphere (7, 20, 26, 27). However, because much of the area of interest is covered by extensive sedimentary basins and inland marginal seas, the geographic coverage of seismic and mantle xenolith observations used in these studies was relatively sparse, providing too limited spatial resolution to adequately address the inherent lateral heterogeneity in the evolution of cratonic lithosphere modification.

Here, we compiled geologic, magnetic, and Cenozoic basin-history datasets to further explore the destruction history of the various cratons along the eastern margin of Asia. We find a striking correlation between magnetic signal, basin thermal-subsidence thickness, and thinned mantle lithosphere, which we interpret together to reflect a signal of weak, deformed continental lithosphere. Our integrated dataset allows for comprehensive analysis of these signals of lithospheric crust–mantle interaction across the cratons of East Asia, including regions obscured from previous studies. We find that the unique Phanerozoic history of the NCC led to focused weakening and lithospheric mantle erosion or delamination. This extended history includes >500 My of circum-craton subduction since the Paleozoic along with the reactivation of major lithosphere-scale faults that acted as conduits to aid this metasomatic weakening, leading to lithospheric mantle removal. Other cratons in East Asia that experienced different geologic histories have not been significantly destroyed by Paleo-Pacific subduction, indicating that one subduction event alone is unlikely to be sufficient to destroy a cratonic mantle root.

## Geological History of the North and South China cratons

The NCC is one of the oldest cratons on Earth, containing Eoarchean crust as old as 3.8 Ga (28). The NCC has remained tectonically stable since the amalgamation of the western and eastern blocks along the Trans North China orogenic belt in the Late Archean–Early Paleoproterozoic (7, 29, 30) until the initiation of subduction zones around cratonic margins in the Early Paleozoic, with associated collisional orogenesis (31, 32) (Fig. 1*B*). Phanerozoic reworking of the NCC differs markedly across the North–South gravity lineament (NSGL) (Fig. 1*C*). To the east of this lineament, the eastern NCC currently has high crustal heat flow (~100 mWm^−2^), abundant seismicity, extensive magmatism, and active extension (7, 33), classifying it as a “modified craton” (1). In contrast, the western NCC, west of the NSGL, has lower heat flow (<40 to 50 mWm^−2^), less seismicity, and less active crustal deformation (7, 33, 34). The eastern NCC is characterized by thin crust (<35 km) and relatively low seismic-velocity lithosphere mantle, while the western NCC is marked by thick crust (>35 km) and an apparently intact high-velocity cratonic mantle root (27).

The SCC in East Asia is separated from the NCC by the Central China Orogenic Belt, also referred to as the Qinling–Dabie–Sulu orogen (Fig. 1*B*). The SCC consists of the Archean-Paleoproterozoic Yangtze block in the northwest and the Cathaysia block in the southeast that coalesced in the Paleoproterozoic (Fig. 1*B*) (26, 35–37). Inland regions of the eastern SCC, east of NSGL, are characterized by highly active crustal deformation and high heat flow (64 to 74 mWm^−2^), as well as low-velocity lithospheric mantle and thin crust (<35 km) (27, 33). The western SCC, mostly covered by the Sichuan basin, is relatively tectonically inactive, has thick crust, an underlying high–velocity mantle root, and low surface heat flow values (average ~ 54 mWm^−2^) (33, 34).

The Tan-Lu fault zone (TLFZ) is a ~2,400-km-long lithosphere-scale strike-slip fault in East Asia that formed during the Triassic-Jurassic collision between the NCC and SCC (Fig. 1) (15, 38). The fault zone cuts the entire NCC with an estimated ~450 km displacement occurring between 240 Ma and 165 Ma, prior to the main destruction of the NCC (7, 15). The TLFZ is characterized by relatively thin lithosphere in its vicinity (~60 km) (39), a low-seismic velocity mantle root (40), and prominent underlying mantle upwelling (41, 42).

## Results

### Analytical Signal Amplitude Model of East Asia.

**Fig. 2.**
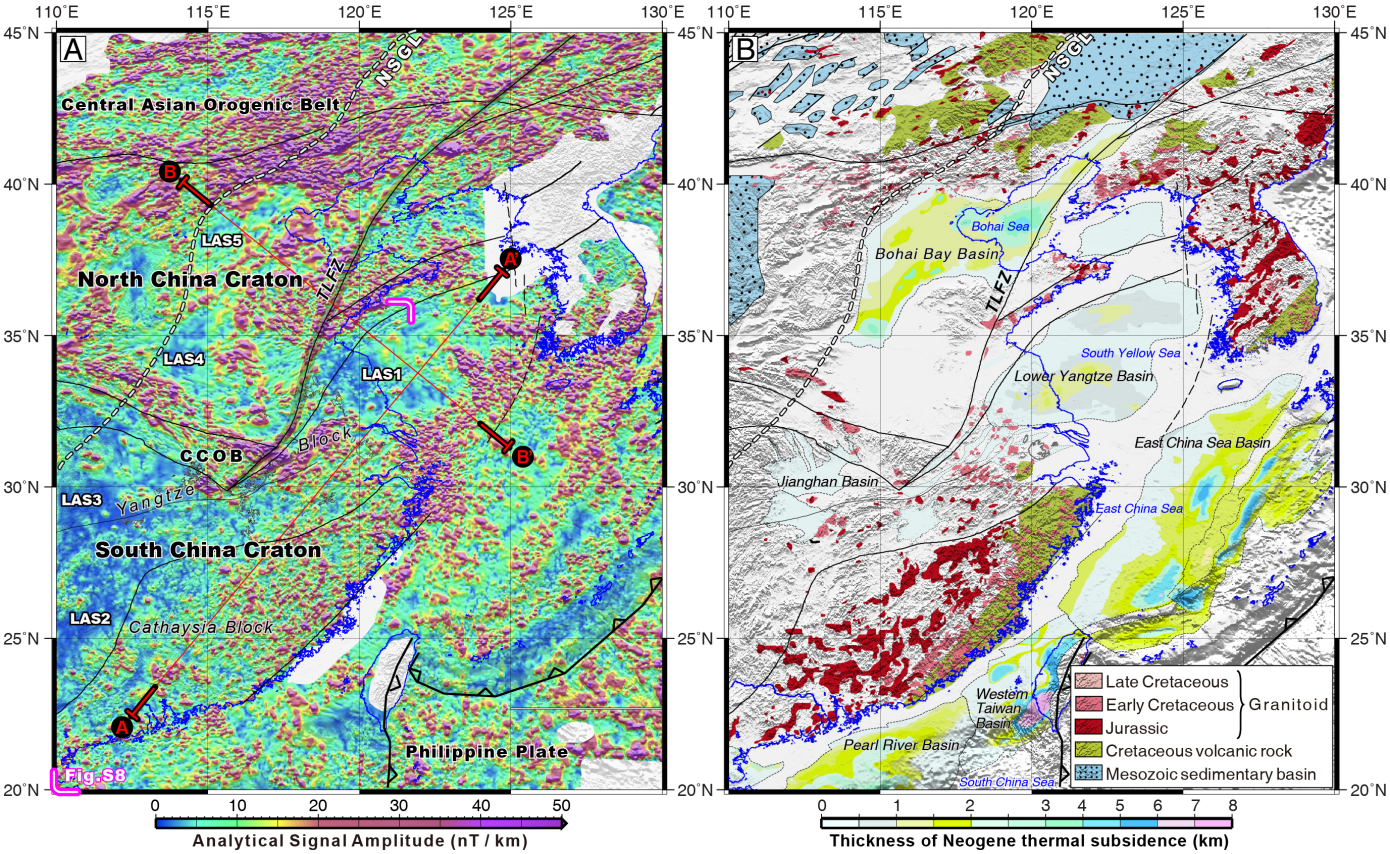
(*A*) Analytical signal amplitude (ASA) model of East Asia calculated from high-resolution total-field magnetic anomalies. The gray domains represent the data gaps. Red sections AA and BB’ are the lines of projection statistics of ASA signal. Statistical treatment of the spatial correlation between the present exposed plutons and the ASA signal indicates that the overlapping percentage is approximately 85% using the Cathaysia block as a case sample (*SI Appendix*, Fig. S5). (*B*) Jurassic-Cretaceous magmatic rocks (7, 19) and the thickness of basinal thermal subsidence during the Late Cenozoic (0 to 24 Ma). The Tan-Lu fault zone (TLFZ) is emphasized by a black transparent strip. The coastline is shown with blue lines.

We constructed a high-resolution crustal scale aeromagnetic dataset across East Asia covering >4,500,000 km^2^ of mixed continental and oceanic regions (Figs. 1*C* and 2*B*). Through reduction to pole transformation, we obtained a high-resolution analytical signal amplitude (ASA) crustal model (*Materials and Methods*; *SI Appendix*, Figs. S1–S4) with a spatial grid interval of 0.01° (~1 km) and 0 to 30 km depth range. The ASA model images magnetic signals that can be interpreted to reflect the distribution and size of magmatic rocks within the crust (12).

The ASA map shows distinct pattern variations in anomalies across East Asia. High-value (>15 nT/km) ASA anomalies are distributed across much of the continent, with especially strong signals concentrated across the NCC and the Central Asian Orogenic Belt (CAOB) to the north (Fig. 2*A*). The CAOB is the largest accretionary orogen in the Phanerozoic that involved the development and collision of numerous arc terranes until its closure in the Permo-Triassic (43). Accordingly, the CAOB is composed of numerous arc plutons that are variably exhumed (44), which correlates with the extensive strip- and spot-like strong ASA anomalies (Fig. 2*A*).

**Fig. 3.**
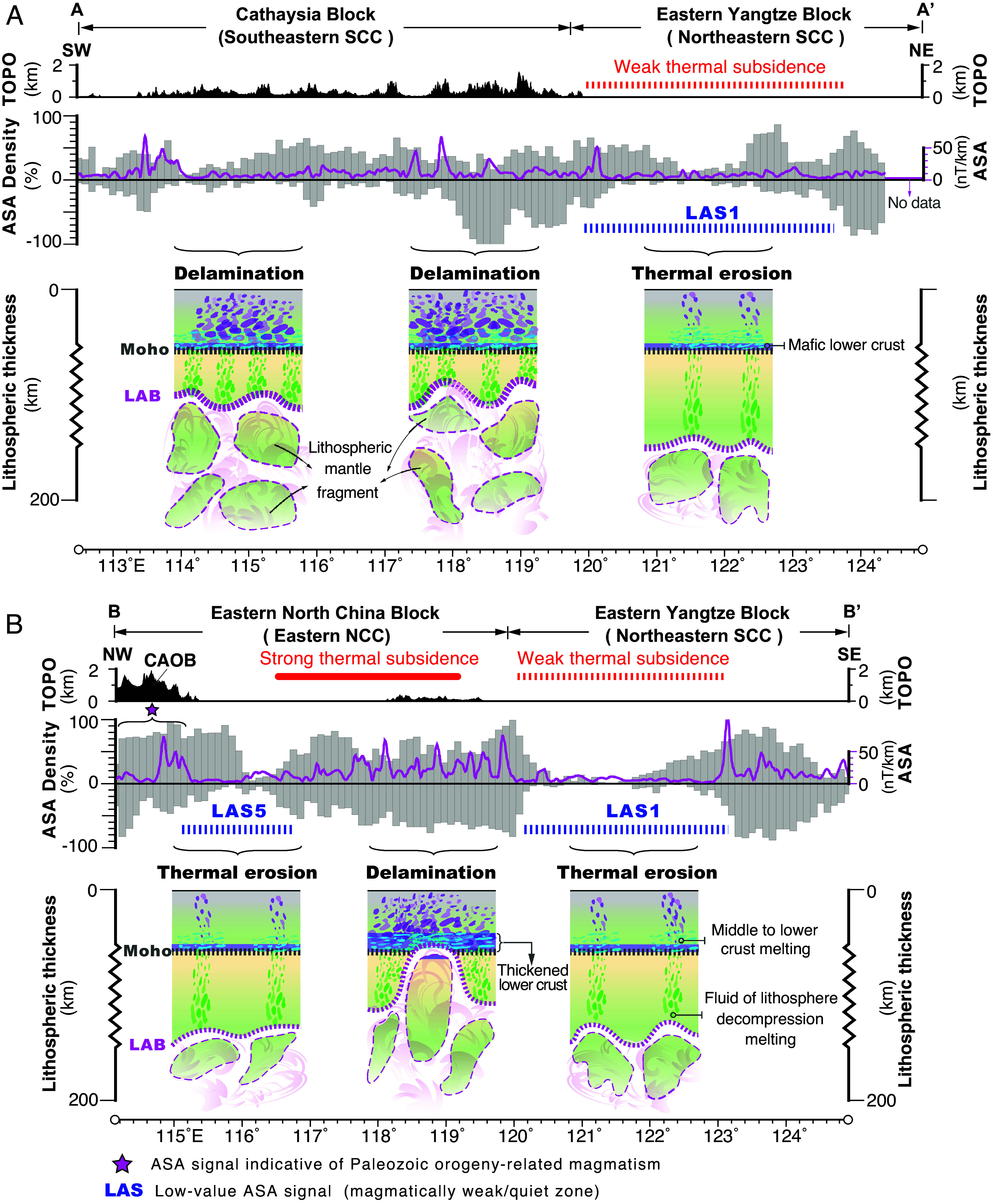
Inferred intrusion density along the A (*A*) and B (*B*) cross sections, parallel and perpendicular, respectively, to the Mesozoic structural orientation of the eastern NCC and Yangtze block of the SCC in Fig. 2*A*. We project the ASA data along sections AA and BB’, with a swath width of two degrees for the projected data (*SI Appendix*, Figs. S6 and S7). The percentage of ASA values is used to indicate the inferred intrusion density. The positive and negative percentages represent the projected ASA value points to the east and west of the section, respectively.

The eastern SCC also displays high ASA values (Fig. 2*A*). There are notable zones of low-value (<15 nT/km) ASA signals (LAS) within the inland continent, such as the zones of LAS1, LAS2, and LAS3 in the SCC, and of LAS4, LAS5, and LAS6 in the NCC (Fig. 2*A*). The ASA signals were projected along NE- and SW-trending profiles to showcase the lateral variations in ASA values across the SCC and NCC (Figs. 2*A* and 3). The southeastern SCC (Cathaysia block) and eastern NCC display strong ASA signals with values of >15 nT/km, whereas the northeastern SCC (eastern Yangtze block) is predominantly characterized by weak ASA anomalies (<15 nT/km) (Figs. 2*A* and 3). The TLFZ is also characterized by predominantly strong ASA anomalies (Fig. 2*A*).

### Mesozoic–Cenozoic Basin History.

The numerous Cenozoic sedimentary basins distributed along the eastern domains of NCC and SCC record two depositional stages: 1) Paleogene extensional rifting with fault-controlled subsidence and 2) Neogene postkinematic thermal subsidence (18, 45–47). We use the Neogene thermal subsidence magnitudes as a proxy of the lithospheric rigidity and thickness of the modified cratons (45, 48) (*Materials and Methods*). Thermal subsidence in these basins all started at ca. 24 Ma, without major latitudinal or longitudinal age trends, as the East Asia lithosphere cooled, contracted, and subsided (18, 47). These basins show pronounced thickness variations across the East Asian lithosphere (Fig. 2*B*). The eastern part of the Yangtze block of the SCC has relatively thin basin deposits (<2 km), whereas the eastern NCC just to the west of the eastern Yangtze block has thick deposits, exceeding 4 km (Fig. 2*B*).

## Discussion

### Multidisciplinary Crustal Signals of Craton Destruction.

Cratonic continental lithosphere with an intact, thermally equilibrated mantle keel is expected to resist voluminous plutonism and large-magnitude thermal basin subsidence and coupled sedimentation (1, 45, 47, 48). Therefore, the generated maps of the extent and magnitude of magmatism and basin sedimentation in this study can be used to evaluate where the cratonic lithospheric keel beneath East Asia was modified or thinned/destroyed (Figs. 2 and 3). Direct observation of the magmatic history across East Asia is often obscured by terrestrial basins and inland seas of the continental shelf. Our ASA maps circumvent this issue and provide an alternative dataset to test in a more comprehensive way (Fig. 2), the extent to which East Asia has been intruded by Mesozoic–Cenozoic magmatism.

We first confirm the spatial correlation between regions of Mesozoic plutons and high-value ASA anomalies. A strong spatial correspondence is observed between widespread exposures of Mesozoic plutons and extensive strip- and spot-like strong ASA signals (Fig. 2). For example, occurrences of Jurassic-Cretaceous plutons widely distributed across the Cathaysia block of the SCC (*SI Appendix*, Fig. S5) and northeastern NCC correlate well with strong ASA anomalies (Fig. 2). For areas without mapped plutons, we interpret that regions of strong ASA signals imply the presence of Mesozoic plutons within the crust. The CAOB region to the north of the NCC similarly shows strong ASA signals that correlate with the voluminous plutonism (Fig. 2*A*) associated with the CAOB evolution (43, 44). Based on the overall correlation of the spotty ASA patterns with Mesozoic plutonism across east China and Paleozoic granitoids in the CAOB, we interpret that the ASA signals reflect subduction-zone magmatism, rather than other igneous processes such as anorogenic diking or plume intrusions that generate more linear or radial ASA anomalies (12, 49).

There is also a strong spatial correlation between ASA signals and basin thermal subsidence. Regions with strong ASA anomalies experienced more pronounced basin thermal subsidence, such as the Bohai Bay and East China Sea basins. Conversely, regions such as the Lower Yangtze and Jianghan basins show lower ASA signals and correspondingly thinner thermal-subsidence basin deposits (Fig. 2).

We posit that variations in Mesozoic magmatism, ASA signals, and the thickness of thermal-subsidence basins predominantly reflect variations in the lithospheric rigidity and thickness due to spatial variations in the extent of modification and thinning of the North and South China continental lithosphere. Regions that have experienced extensive intrusion by Mesozoic magmatism, observed via surface geology and corresponding ASA signals, reflect thinner and weaker mantle lithosphere at the time of plutonism that allowed for more voluminous melting at shallower pressures (50, 51). The distribution of plutons and strong ASA signals further demarcates the extent of the upper-plate lithosphere that was influenced by subduction-zone processes, including significant hydration and metasomatism (6, 7). The thicker thermal-subsidence basins correspond to lithosphere that was more weakened or thinned prior to the initiation of thermal subsidence at ca. 24 Ma (18, 45, 47, 48).

Taken together, the map pattern variations in plutonism and thermal-subsidence basin records serve as proxies for deciphering the heterogeneous modification to the East Asian continental lithosphere. This timing of lithospheric modification is bracketed between the Mesozoic, when spatially variable plutonism occurred, and the Neogene, when spatially variable thermal-subsidence basins developed. Based on the regional geologic history, the variable modification of the East Asian continental lithosphere most likely occurred during the Mesozoic west-dipping subduction of the Paleo-Pacific plate (6, 18). For example, the eastern NCC, which has long been interpreted to have experienced mantle root destruction in Mesozoic times (7, 52, 53), displays strong ASA anomalies, voluminous Mesozoic plutonism, and widespread basins with thermal-subsidence thicknesses of >4 km.

To further validate this hypothesis, we compared these proxies of lithosphere modification with high-resolution tomographic imaging across the continental SCC of ref. 14 (*SI Appendix*, Fig. S8). The western Yangtze block of the SCC exhibits limited Mesozoic plutonism, weak ASA anomalies, and thin basins, which correspond to a seismic high-velocity mantle root at depth. Conversely, the southeastern Yangtze and Cathaysia blocks of the SCC display strong ASA signals, voluminous Mesozoic magmatism, and relatively thick basin deposits above a low-velocity lithospheric mantle (Figs. 2 and 3). This correlation confirms that the thickness of an intact mantle root is negatively correlated with proxies of Mesozoic magmatism, ASA anomalies, and basin thickness in East Asia.

### Craton Destruction Through Protracted Metasomatic Weakening and Lithospheric Thickening.

Cratonic mantle roots are refractory and dehydrated, which explains their persistence and resilience over billion-year timescales (2, 3, 54). Therefore, the erosion or detachment of a mantle root requires thermo-mechanical or chemical weakening (55, 56). Most models for the destruction of the NCC emphasize the involvement of a single subduction event as the first-order control on craton modification and destruction, exemplified by the west-dipping subduction of the Paleo-Pacific (Izanagi) plate (6, 7, 24, 57). Subduction of hydrated oceanic crust releases fluids and volatiles into the overlying mantle lithosphere (58, 59). Such hydration can lead to hydroweakening and metasomatism of the mantle lithosphere (60), which allows for subsequent erosion or detachment of a cratonic lithospheric mantle root (55, 56). However, if the westward subduction of the Paleo-Pacific plate hydrated and uniquely weakened the NCC, it is predicted that this process should have similarly modified and destroyed the SCC based on its spatial position west of the subduction trench (26). Any spatially variable modification of either craton implies intrinsic differences in the strength and durability of the cratonic mantle lithosphere between the two cratons.

The various proxies discussed above show strongly contrasting extents of lithosphere modification between the NCC and SCC, consistent with the results from tomographic imaging (27) and geological investigation (26). Within the SCC, significant variations of lithospheric modification are observed, with the western Yangtze block interpreted to have intact thick mantle lithosphere, whereas the eastern Yangtze block, just east of the NCC, shows evidence for only modest lithospheric modification and thinning (Fig. 3) (14, 26). These observations suggest that the lithospheric mantle beneath much of the easternmost Asian margin near the subduction trench was significantly modified by the westward Paleo-Pacific subduction (Figs. 1 and 2) (7, 10, 26). Although subduction-zone processes, including Mesozoic plutonism and lithospheric thinning, impacted the East Asian lithosphere from the Pacific trench to as far inboard as the NSGL (13, 18, 61) (Fig. 2), there are several distinct regions in the SCC that do not appear to have experienced significant lithospheric mantle weakening (labeled LAS1, 2, and 3) (Fig. 3) based on thin Neogene thermal-subsidence basin deposits, an absence of a strong ASA signal, and limited Mesozoic magmatism (Fig. 2). Adjacent to these regions where the lithosphere appears to remain intact, at a similar distance from the Pacific subduction trench, the lithosphere beneath the NCC appears to have been strongly modified (Figs. 2*A* and 3). Taken together, these results indicate that subduction of the Paleo-Pacific oceanic slab alone does not account for the above observations, including the presence of regions of intact or limited thinned mantle lithosphere, or partially healed lithosphere (14) in the SCC. Therefore, we propose that the strong spatial variability in the extent of modification of lithospheric mantle across North and South China should reflect inherited structural and compositional differences in the pre-Pacific-subduction mantle lithosphere.

**Fig. 4.**
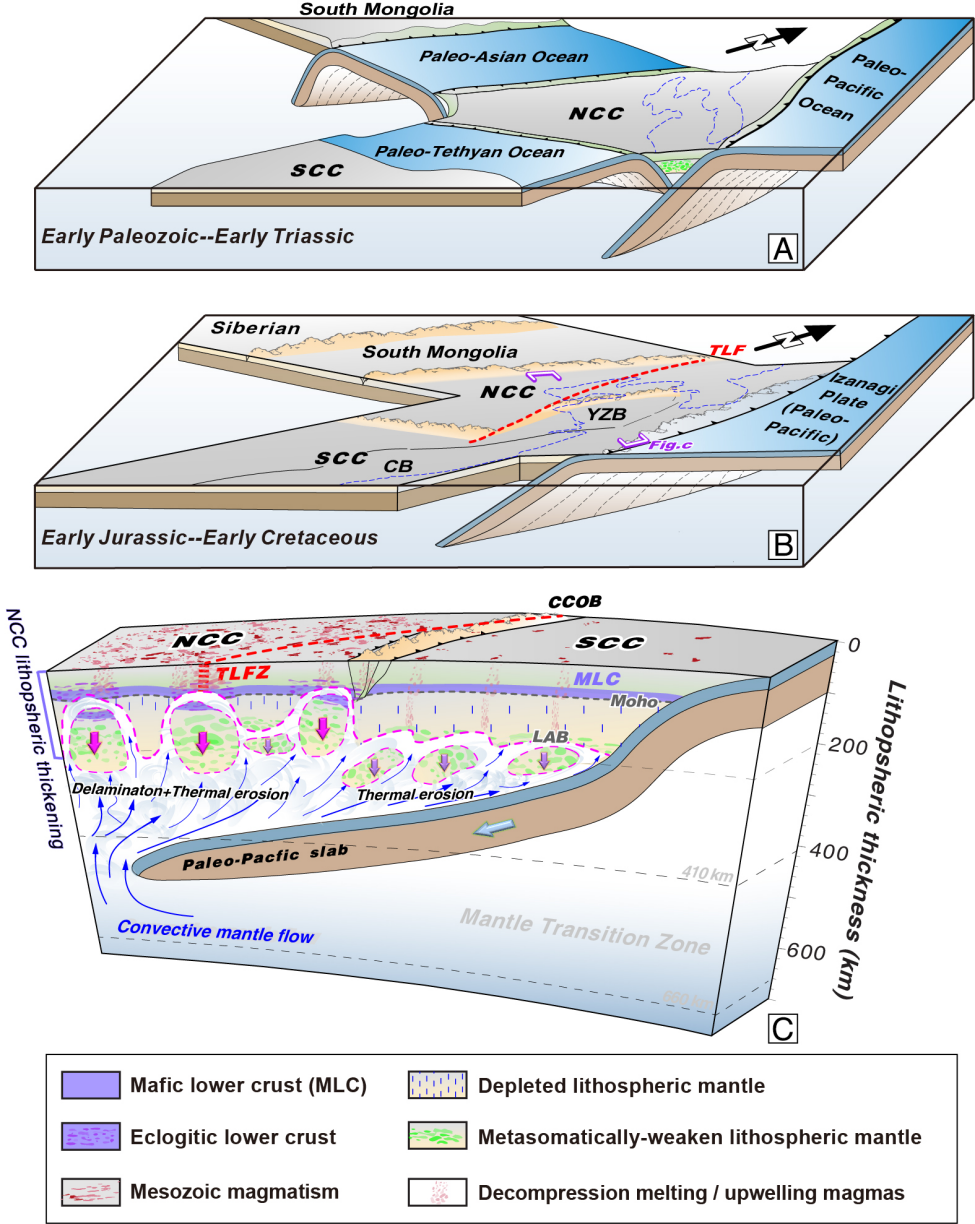
A model for the lithospheric modification for eastern NCC and Yangtze block of the SCC, with the model figure inspired by ref. 32. (*A*) Early Paleozoic to Early Triassic circum-NCC subduction system. (*B*) Early Jurassic-Early Cretaceous Paleo-Pacific (Izanagi) subduction system. (*C*) Differential mode of lithospheric modification and thinning for the eastern NCC and northeastern SCC. YZB, Yangtze block; CB, Cathaysia block; TLFZ, Tan-Lu fault zone; CCOB, Central China orogenic belt.

The Phanerozoic tectonic history of the NCC involved protracted oceanic subduction and subsequent collisional orogenies along its margins, including the south-dipping Ordovician-Triassic Paleo-Asian Ocean and Carboniferous-Jurassic Mongol-Okhotsk Ocean to the north, the north-dipping Carboniferous-Triassic Paleo-Tethyan Ocean to the south, and the Jurassic-Early Cretaceous Paleo-Pacific Ocean to the east (6, 32, 60, 62) (Fig. 4 *A* and *B*). The SCC was not affected by such protracted subduction in the Phanerozoic and remained surrounded by passive margins until the Mesozoic (28). The duration of cumulative subduction beneath the NCC amounts to >500 My, whereas the eastern SCC was only modified by the discrete Paleo-Pacific subduction event that has been acting for less than ca. 200 My (7, 24, 61). Overall, during the extended period of circum-continent subduction experienced by the NCC, its mantle keel would have potentially been exposed to fluid flux and volatile release from the various subducting slabs (63–65) up to three to five times greater than the eastern SCC root, which experienced subduction over a much shorter duration (Figs. 3 and 4). We suggest that due to an unusual set of circumstances, the NCC experienced more continuous circum-craton subduction, cratonic mantle root metasomatism and associated hydroweakening, plus longer-lived subduction-collision-derived lithospheric thickening than any other Archean craton during the Phanerozoic (6, 66) (Fig. 1*A*). The Paleozoic–Mesozoic circum-NCC subduction systems led to collisional orogenies and crustal thickening across the NCC (67, 68). Such crustal thickening would have led to eclogitazation of the lower crust (69), priming it for wholesale lithospheric delamination and catastrophic craton destruction subsequent to thermal removal of lowermost portions of the lithospheric mantle root (6).

Paleo-Pacific subduction and rollback-derived mantle convection impacted the mantle lithosphere of the eastern NCC and SCC that were closest to the subduction trench, but this subduction alone was not able to modify the cratonic mantle within the continental interior, as evidenced by the intact mantle root beneath the western NCC and SCC (Fig. 2) (10, 14). Instead, circum-continent subduction and related lithospheric metasomatic weakening and collisional thickening played a key role in the eastern NCC’s wholesale lithosphere removal via delamination coupled with thermal erosion. In contrast, only limited lithospheric thinning without catastrophic craton destruction occurred in the northeastern SCC (especially the eastern Yangtze block portion) due to the absence of circum-continent lithospheric weakening and collisional thickening (Fig. 4*C*).

### Evolution, Destruction, and Survival of Cratonic Mantle Through Earth History.

Our new map of the spatial variability of lithospheric modification of the North and South China cratons confirms that the activity of a single subduction system with less than ca. 200 My duration (7, 13, 70) may not be the sole factor involved in the catastrophic destruction of cratonic mantle roots (Fig. 2). Indeed, subduction systems were common along the peripheries of long-lived stable cratons since the Paleoproterozoic (71–74). A typical single-sided subduction involving flat-slab events and subsequent rollback may partially modify the margins of cratons, as suggested for the South China, Amazonian, and Wyoming cratons around the Pacific Ocean, which represent a relatively significant portion of Earth’s cratons being actively modified in the past 100 to 200 My (66, 70, 75). However, if subduction systems consistently destroyed even just the marginal regions of cratonic lithosphere, the geometry of cratons would be substantially modified during the collision and growth of most supercontinent cycles over the past >2.0 Gy, which is at odds with our ability to reconstruct supercontinent configurations with conjugate plate margins (76–78) and the inferred importance of lateral accretion processes in the overall growth of cratons (1). Therefore, for a single-sided, short-duration (<200 My) subduction, its modification of the cratonic margins is unlikely to significantly destroy cratonic mantle roots.

Our study emphasizes that very unique circumstances are required for what must be a relatively rare process—craton root destruction—which corroborates the long-term survival of most cratons on Earth. The unique nature of the protracted circum-cratonic subduction/collision systems that impacted the NCC over >500 My gradually metasomatized, weakened, and potentially thickened its mantle keel. This showcases the pronounced differences between the NCC and other cratons such as the SCC, Wyoming, and Amazonian cratons, which were partially modified by subduction-zone processes (66, 79), but such processes have yet to lead to complete destruction of their roots (1). For example, new datasets show that the Wyoming craton was less impacted by lithospheric erosion than previously thought, with significant modification and thinning of its mantle root constrained to the western extent of the Wyoming craton (80). The modified western Wyoming craton serves as a “crumple zone” to focus deformation (81) such as the Cretaceous–Paleogene Sevier/Laramide shortening and Cenozoic Basin and Range extension, shielding the core of the craton from deformation. It is unlikely that single-sided subduction systems acting alone could efficiently destroy cratonic mantle roots globally in the short time since the Mesozoic, because if this were the case, approximately one-third to one-half of Earth’s cratons would have been modified in this way since 200 Ma (Fig. 1*A*). This is at odds with the recognition that cratons survive over billions of years of Earth history (1). Flat-slab subduction processes have been proposed to be important for craton destruction (70, 82, 83), but they are common along many subduction zones along East Asia and the western Americas, including the entire strike length of the South American Andes (7, 70). Flat-slab subduction therefore cannot consistently destroy cratonic mantle, which may partially reflect the short timescales (<50 My) over which shallow subduction occurs, controlled by the time when basaltic oceanic crust remains metastable before metamorphosing into dense, negatively buoyant eclogite (84, 85). Furthermore, although the global comparisons further reinforce the hypothesis that large-scale craton destruction is generated only in cases of prolonged circum-continent subduction, here we emphasize that the Pacific subduction process served as the final trigger for the craton’s destruction.

With time, plume-driven recratonization (11–13) and subduction-associated lithospheric relamination (14, 15) are likely two important processes to heal modified cratonic lithosphere. The persistence of cratonic lithosphere may occur because partially modified lithospheric mantle can potentially heal over a period of ~100 to 300 My (13, 83–89). For example, tomographic imaging across the southeastern SCC reveals seismically fast Early Cretaceous-delaminated mantle lithosphere at ~90 to 120 km depth that may be actively relaminating (14) (Fig. 3). This offers an insight into why a single subduction event rarely destroys entire cratons throughout Earth history. Cratonic mantle keels may heal after the cessation of subduction (15).

In summary, we show how the heterogeneous destruction of the mantle root of the NCC was driven by a history of continuous circum-craton subduction and orogeny that weakened the NCC mantle through protracted tectonic attrition over >500 My via mantle metasomatism, hydration, and lithospheric thickening. There were no major hiatuses for the cratonic mantle to heal via relamination (14, 86, 90) or plume residue accretion (11, 13), and thus the NCC mantle was primed for wholesale destruction during west-dipping Paleo-Pacific subduction. This attrition hypothesis suggests that craton destruction in East Asia was unique, requiring unusual circumstances and offering a rationale for why most cratons are not destroyed throughout Earth history.

## Materials and Methods

### Aeromagnetic Data Compiling and Processing.

A new regional total-field magnetic map of East China was constructed to examine the crustal magnetic character. Aeromagnetic data within and around the NCC were previously measured to aid in petroleum and mineral exploration before 2010s by the China Aero Geophysical Survey and Remote Sensing Center for Natural Resources (AGRS) (91) (*SI Appendix*, Fig. S1), China Geological Survey. The aeromagnetic compilation combines more than 200 aeromagnetic surveys, flown between 1960s and 2010s and with variable flight-line spacing (*SI Appendix*, Fig. S1). The original data were processed following previously reported protocols at the China Geological Survey-AGRS (Beijing) (91). To fill the aeromagnetic data gaps, the dataset of CCOP (Geological Survey of Japan and Coordinating Committee for Coastal and Offshore Geoscience Programmes in East and Southeast Asia) with 1-km grid and 1-km altitude) was merged into the AGRS aeromagnetic data (92) (*SI Appendix*, Fig. S1). To be consistent with the CCOP dataset, AGRS aeromagnetic dataset was first processed by upward-continuation to 1 km and then by resampling at 1-km grid, which was all integrated into the Geosoft Oasis Montaj software package (https://www.geosoft.com/products/oasis-montaj). The total-field aeromagnetic anomalies within and around Eastern China were finally compiled with 1-km resolution at 1-km altitude. Finally, we stitched reprocessed AGRS and CCOP magnetic datasets together and then executed the differential reduction to the pole (93) to accurately relocate magnetic source locations and boundaries of East China and its environs (*SI Appendix*, Fig. S2).

To better illustrate the magmatism from magnetic anomalies, we calculated 3D ASAs, which are defined simply as

$$|A(x,y)|=\sqrt{(\partial M/\partial x{)}^{2}+(\partial M/\partial y{)}^{2}+(\partial M/\partial z{)}^{2}},$$

where M is the total-field magnetic anomaly. Essentially equivalent to the total magnetic gradient, ASAs are independent of the inclinations and declinations of the source magnetizations and the Earth’s magnetic field if the magnetic contacts are nearly vertical (94, 95). Unlike original magnetic anomalies, ASAs are always positive, with peaks tending to be located directly above the magnetic sources and/or their contacts. This makes the geological interpretation of ASAs much more straightforward (*SI Appendix*, Figs. S3 and S4).

Moreover, we compiled 346,796 detailed rock-magnetic susceptibility measurements from East China (*SI Appendix*, Fig. S9). The measured susceptibility concentrated in the igneous suites ranges from 20 × 10^−5^ to 10,000 × 10^−5^ SI, with high mean values of more than 1,000 × 10^−5^ SI (*SI Appendix*, Fig. S9), indicating that the high-value ASA signals can indeed be explained by magmatic plutons, corroborated by surface exposures (Fig. 2*B*).

### Neogene Thermal Subsidence Thickness.

We compiled two-dimensional seismic-reflection transects across the East China continental shelf (96) released to us by China National Offshore Oil Corporation for research purposes, totaling >930,000 kilometers, to examine the thickness of Neogene thermal subsidence on the East Asian continents. We also synthesized previously published thermal-subsidence data offshore to construct a comprehensive database of Neogene thermal subsidence across eastern China (97).

The magnitude of thermal subsidence in sedimentary basin varies as a function of lithospheric rigidity and lithospheric thickness, including how much the lithosphere has been stretched and thinned (47). This sets up the prediction that the modified lithosphere, which has been changed from an initial cratonic lithospheric state, should accommodate more basin subsidence than its intact counterpart.

The NCC, which is believed to have been destroyed since the Mesozoic, has thicker Neogene subsidence basins when compared with the SCC. Most of the South China regions have thinner subsidence basins, implying less impacted lithosphere when compared with North China, except in the eastern marginal region, where thicker subsidence basins are observed and correlate with lithosphere that was most modified by recent west-dipping subduction of the Pacific Ocean (18, 46). That is, the thinner lithosphere of the eastern NCC allows for more significant lithospheric extension and thermal subsidence, whereas the thicker intact Yangtze lithosphere resists such basin subsidence (Fig. 2*B*).

## Supplementary Material

Appendix 01 (PDF)

Dataset S01 (TXT)

## Data Availability

All study data are included in the article and/or supporting information.
