# Supplementary material for: Protracted circum-continent subduction: A mechanism for craton destruction and a rationale for craton longevity
Source: Proc Natl Acad Sci U S A. 2025 Aug 7;122(32):e2502618122. doi: 10.1073/pnas.2502618122 (PMC12358890; doi:10.1073/pnas.2502618122)
Supplement: Supplementary file 1 — Appendix 01 (PDF) [file pnas.2502618122.sapp.pdf]

PNAS

Supporting Information for

Protracted circum-continent subduction: A mechanism for craton destruction and a rationale for craton longevity

Xi Xu*, Andrew V. Zuza, Lijun Liu, Weilin Zhu, Yanyun Sun, Baodi Wang*, Xingtao Kuang, Song Han, Xuanjie Zhang, Wan Zhang, Xiaowei Fu, D. Graham Pearson, and Jingao Liu*

*Corresponding author: Xi Xu, Baodi Wang, and Jingao Liu

Email:

xuxigeo@gmail.com, lexus.phd@gmail.com (Xi Xu);

baodiwang@163.com (Baodi Wang);

jingao@cugb.edu.cn (Jingao Liu).

This PDF file includes:

Figures S1 to S9

SI References

Other supporting materials for this manuscript include the following:

Datasets S1

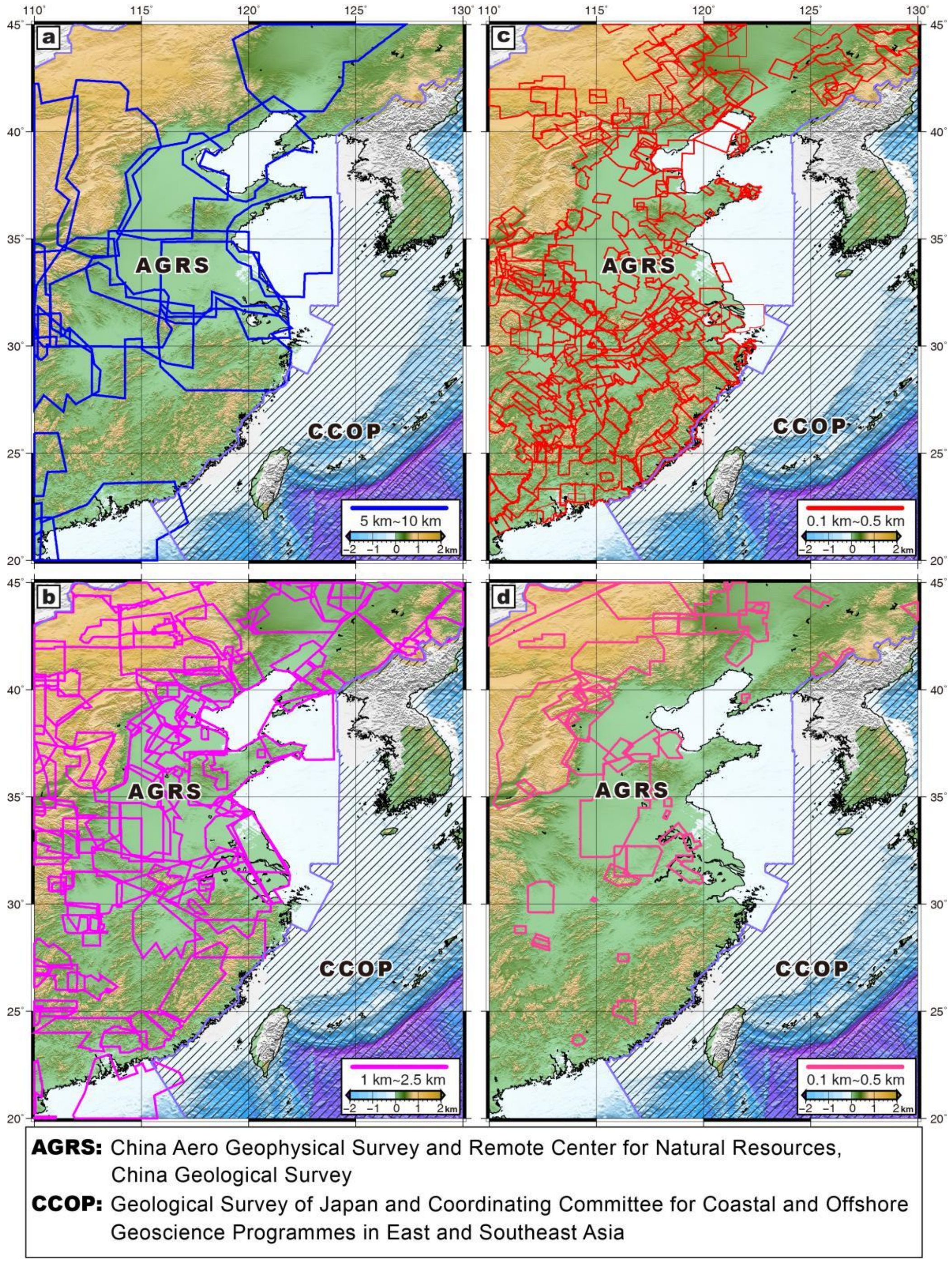

Fig. S1 AGRS aeromagnetic and CCOP magnetic datasets across the East Asian. Colored polygons show different flight-line spacing for compiled data (**a, b, c** and **d**). The two digital data grids are stitched together along the blue line. The AGRS aeromagnetic compilation for this study region combines more than 200 aeromagnetic surveys, flown between 1960 and 2011 and varying in flight-line spacing of 0.5/1/2.5/5/10 km and elevation ranging from 0.6 to 3 km following the terrain. The non-colored regions indicate the grid gaps. Data sources: China Geological Survey Geocloud Database (http://geocloud.cgs.gov.cn/) and refs. (1, 2, 3, 4).

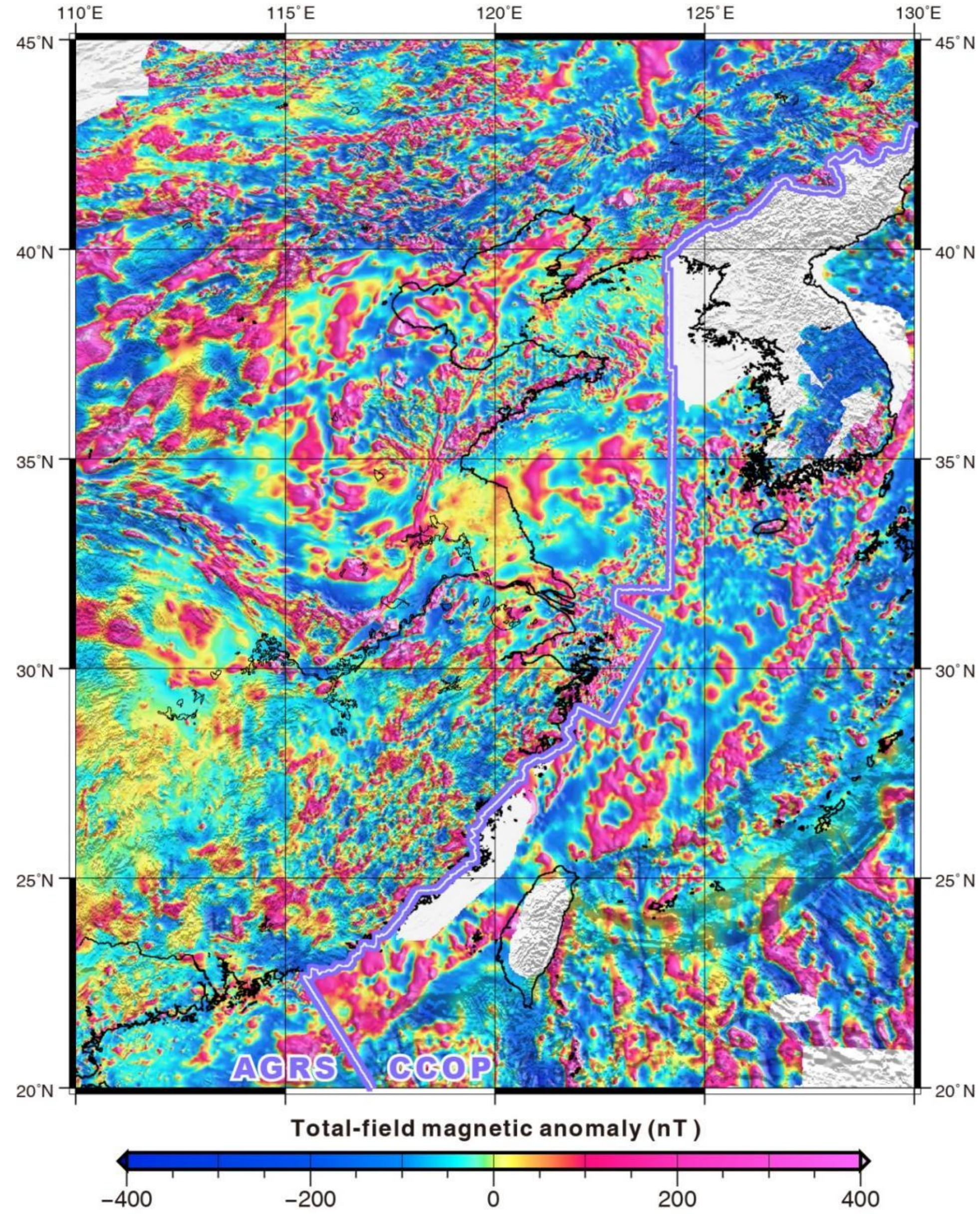

Fig. S2 Differential reduction to a pole map of the magnetic anomalies for the East Asian, overlain on the hillshade DEM model. The warm red and cool blue colors depict magnetic highs and lows, respectively. The light purple and white curve line represents the boundary along which the AGRS and CCOP datasets merged together. AGRS, China Aero Geophysical Survey and Remote Center for Natural Resources, China Geological Survey; CCOP, Geological Survey of Japan and Coordinating Committee for Coastal and Offshore Geoscience Programms in East and Southeast Asia. Gray topographic zones represent the regions absent of magnetic data. The high- resolution AGRS aeromagnetic data set with a 1 km × 1 km grid could be used for research purposes via the application in the China Geological Survey Geocloud Database (http://geocloud.cgs.gov.cn/), or digitized from the high-resolution aeromagnetic map of the China mainland (1).

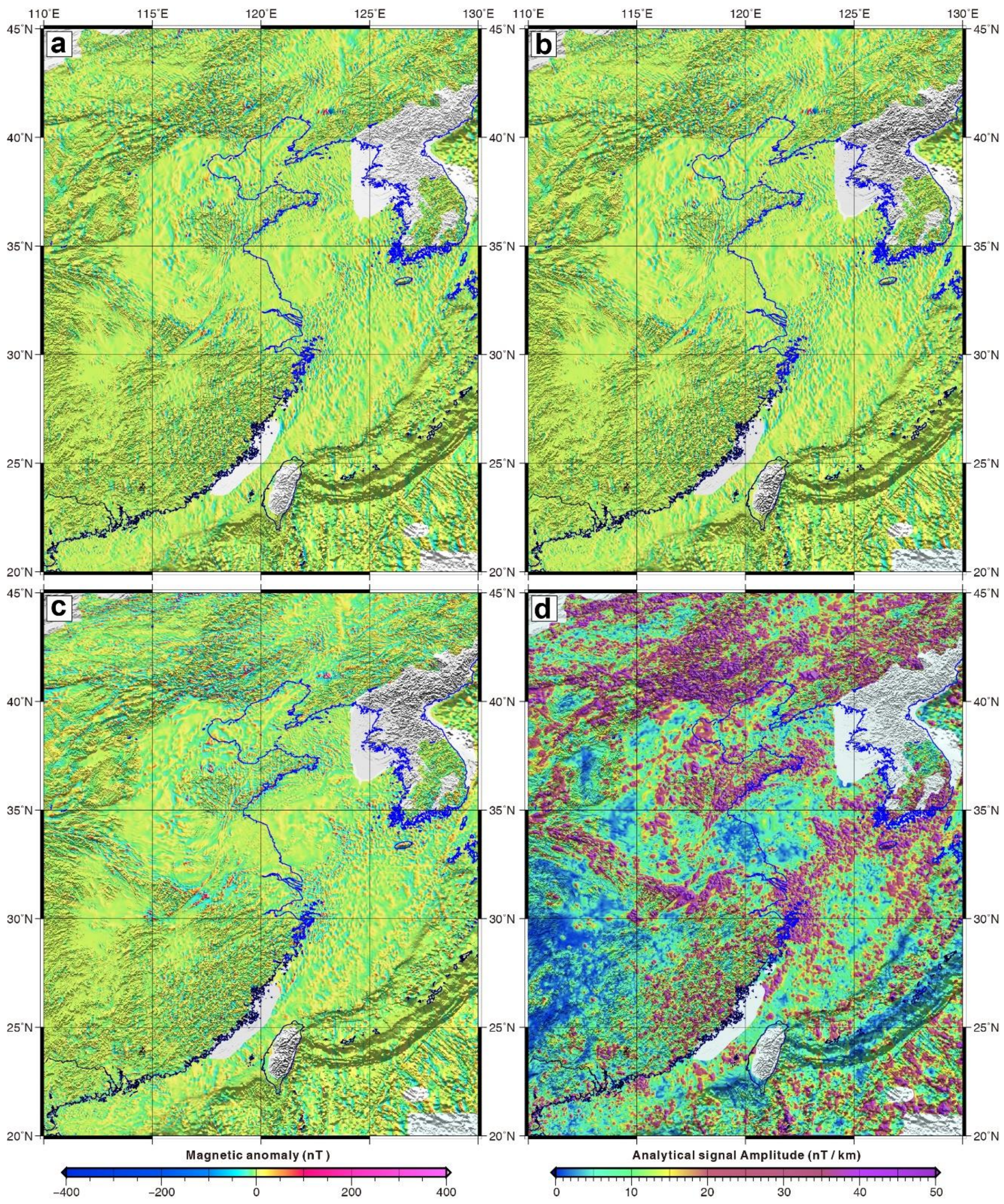

Fig. S3 Processed results of analytic signal amplitude of the magnetic anomalies. (**a**) Horizonal-X, (**b**) Horizontal-Y, (**c**) Vertical-Z derivatives and (**d**) Analytical Signal Amplitude (ASA) are calculated from the total-field magnetic anomaly of differential reduction to pole (Fig. S2). Overlapped on the map are costline contours of 0 m with blue color. Gray topographic zones represent the regions absent of magnetic data.

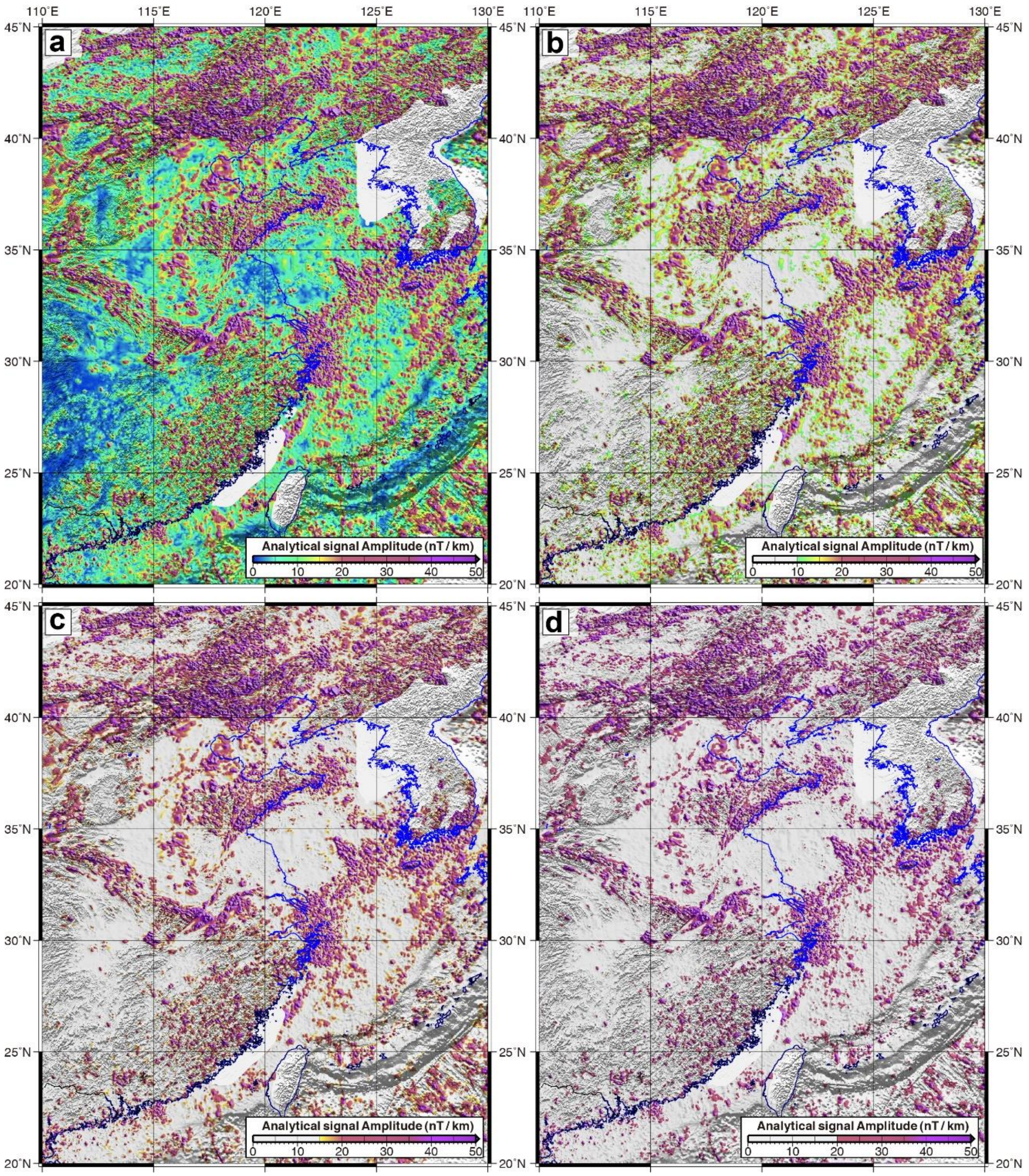

Fig. S4 Maps of analytic signals with different color-value ranges, (**a**) 0-50 nT/km, (**b**) 10-50 nT/km, (**c**) 15-50 nT/km, and (**d**) 20-50 nT/km. The locations of the maxima and the shape of this signal can then be used to find magnetic body edges and volcanic rock bodies. Overlapped on the map are costline contours of 0 m with blue color. Gray topographic zones represent the regions absent of magnetic data.

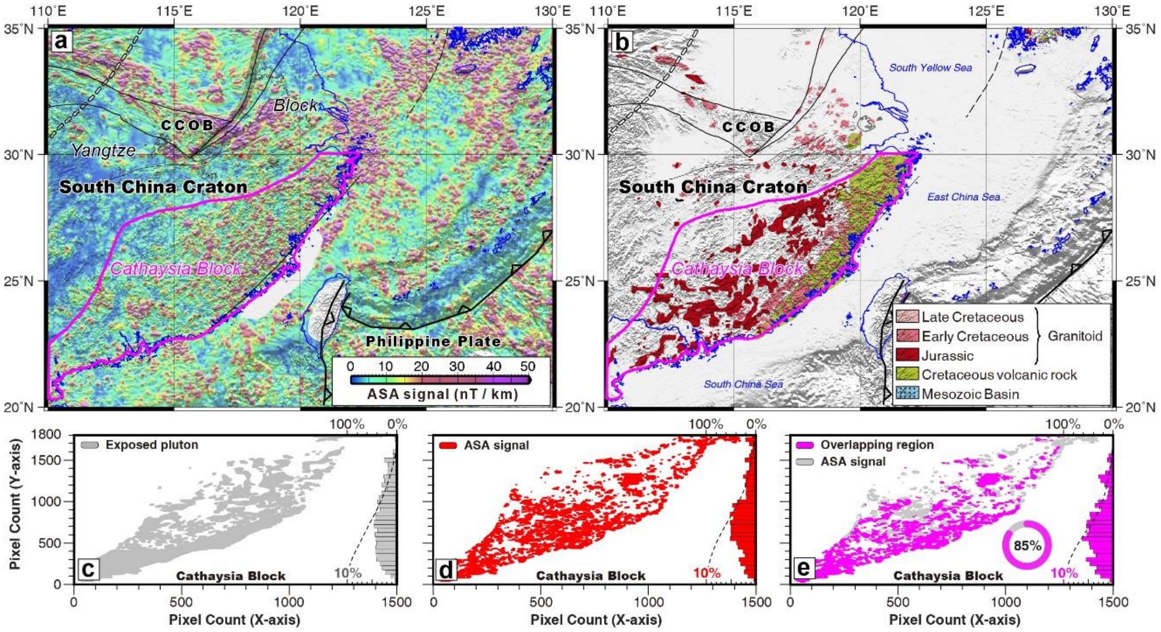

Fig. S5 Statistical treatment of the spatial correlation between the present exposed plutons (**a)** and the ASA signal (**b)** in the Cathaysia block. Distribution and statistics of pixel count of exposed plutons (**c)**, ASA signal (**d**), and overlapping region (**e**). The overlapping regions indicate the spatial location intersection between the ASA signals and exposed plutons. The overlapping percentage is approximately 85% using the Cathaysia block as a case sample for the statistics, where is outlined by pink lines. The ASA threshold value for statistical treatment is 15 nT/km. All the values of ASA data points are bigger than 15 nT/km. Gray topographic zones represent the regions absent of magnetic data.

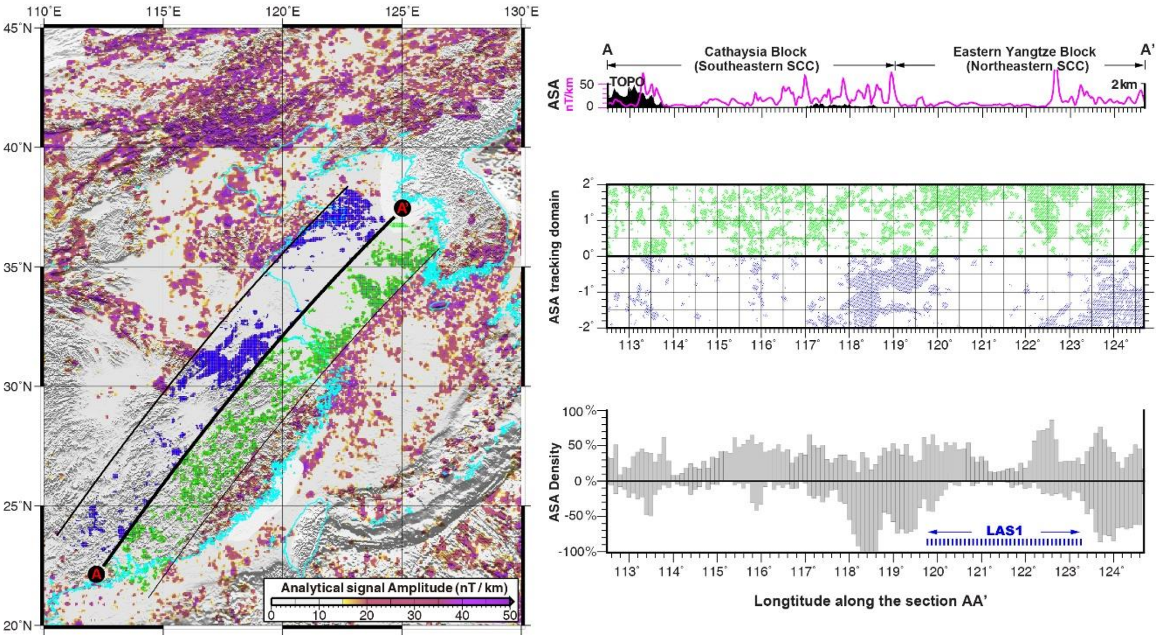

Fig. S6 Projection of analytical signal amplitude (ASA) along the section AA'. We specify the width of two degrees to control for the projected points. These blue and green ASA points are projected on the section AA'. All the ASA points are read to calculate histogram based on the bin-width of 0.1 degree. The percentage of ASA values is used to indicate the inferred intrusion density. The positive and negative percentage present the projected ASA value points to the east and west of the section AA', respectively. Overlapped on the topographic map are costline contours of 0 m with cyan color. Low-value (<15 nT/km) ASA signal (LAS1) is labled in Fig. 2A. Gray topographic zones represent the regions absent of magnetic data.

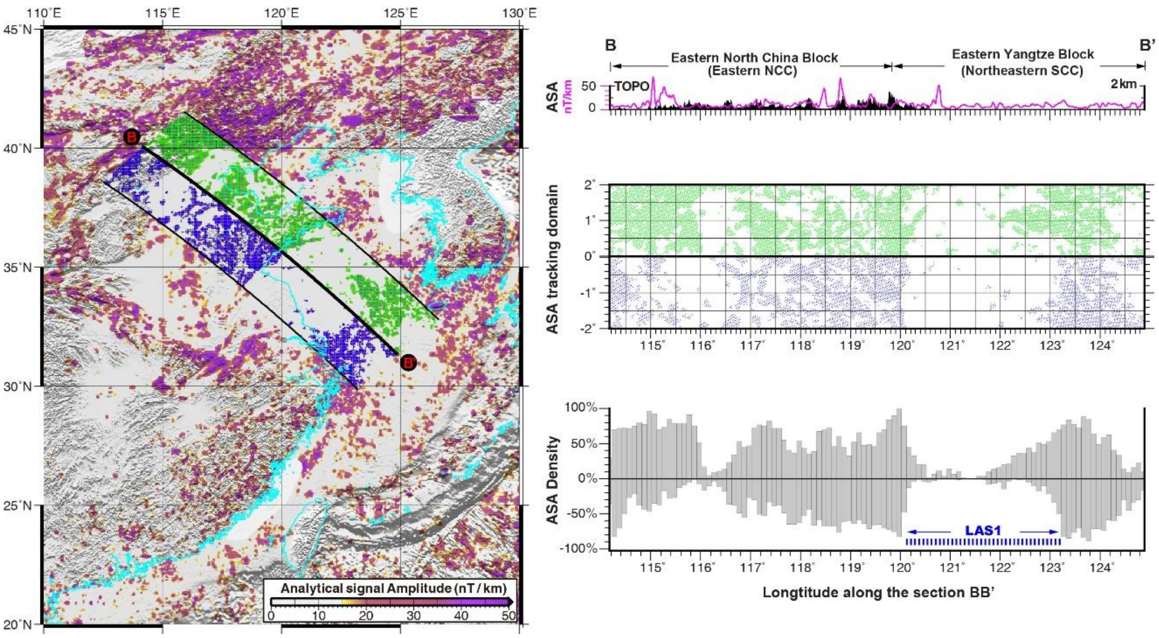

Fig. S7 Projection of analytical signal amplitude (ASA) along the section BB'. We specify the width of two degrees to control for the projected points. These blue and green ASA points are projected on the section BB'. All the ASA points are read to calculate histogram based on the bin-width of 0.1 degree. The percentage of ASA values is used to indicate the inferred intrusion density. The positive and negative percentage present the projected ASA value points to the east and west of the section BB', respectively. Overlapped on the topographic map are costline contours of 0 m with cyan color. LAS1 is labled in Fig. 2A. Gray topographic zones represent the regions absent of magnetic data.

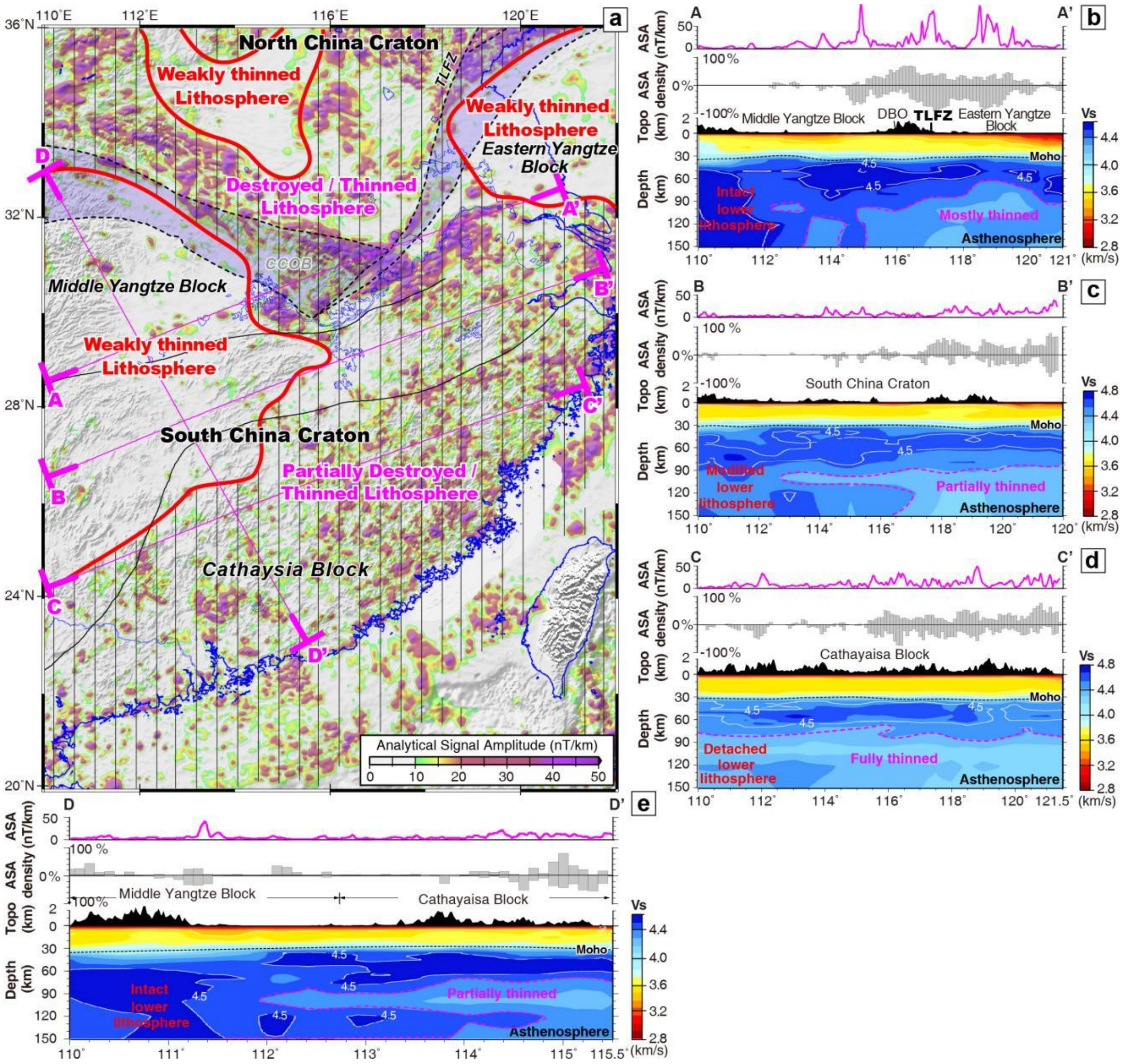

Fig. S8 Crustal-scale ASA model of East China (**a**). Cross-sections of correlation between the crustal ASA model and lithospheric Vs model (5) are shown along profiles AA' **b**, BB' **c**, CC' **d**, and DD' **e** with positions shown in Fig. **S8a**, respectively. The domains of intact lithosphere across East China are shown in Fig. **S8a**. The thick red lines in Fig. **S8a** denote the regions of destroyed and/or thinned lithosphere. Gray topographic zones represent the regions absent of magnetic data.

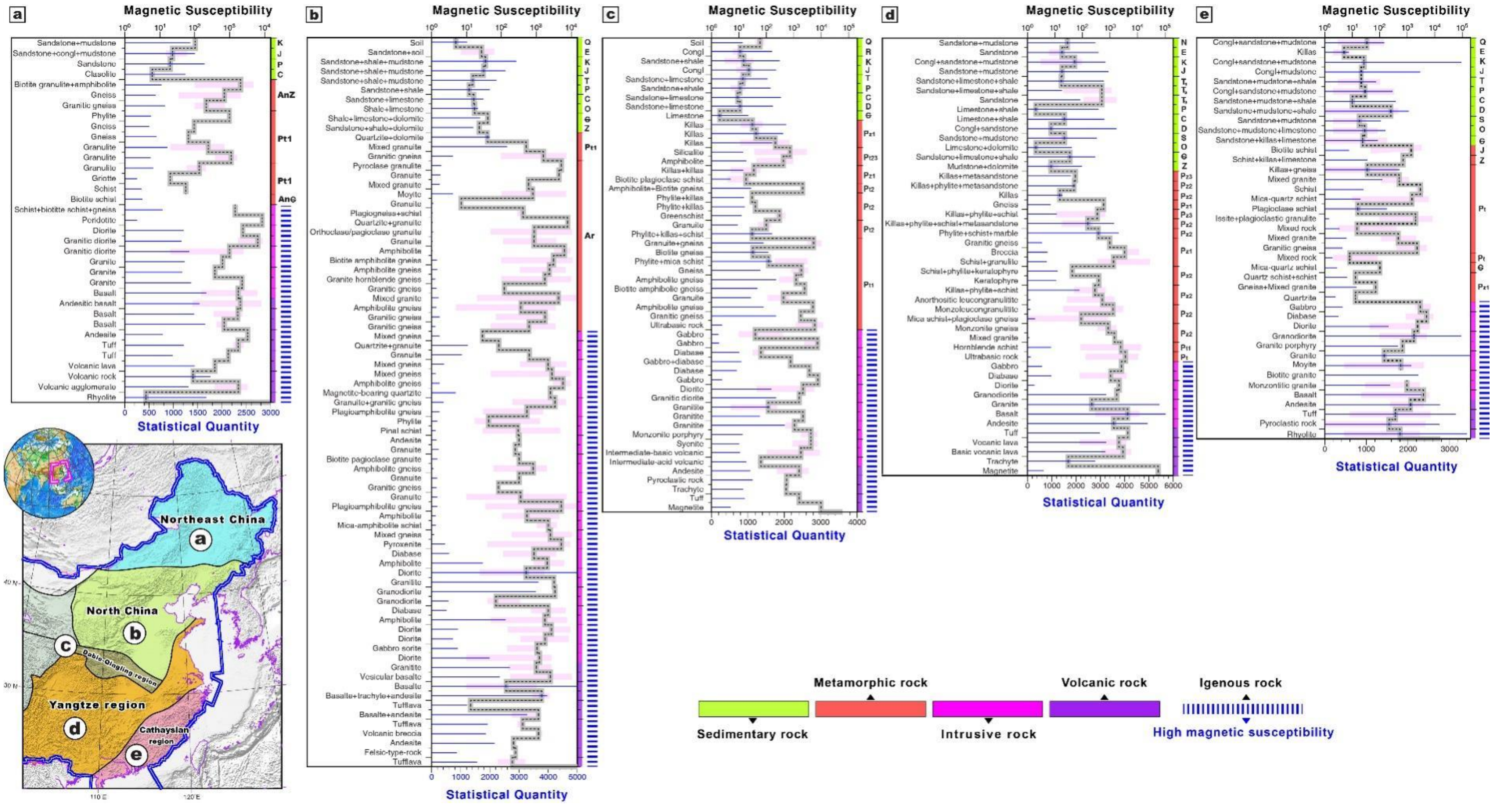

Fig. S9 Statistics of magnetic susceptibility of surface exposure and drilled-core rocks including sedimentary, metamorphic, intrusive, volcanic and other rocks within the Northeast China (**a**), North China (**b**), Qinlin-Dabie region (**c**), Yangtze region (**d**) and Cathaysian region (**e**) (2, 3). SS, Sandstone; MS, Mudstone; CM, Conglomerate; CB, Carbonatite; Inde-MES, indefinite metamorphic rock; Inde-gneiss, indefinite gneiss; Inde-granite, indefinite granite; Inde-rock, indefinite intrusive rock.

SI References

1. S. Xiong et al., Aeromagnetic Series Map of China's Mainland and its Specification: Scale: 1: 2,500,000 (Geological Publishing House, 2013).

2. S. Xiong, J. Tong, Y. Ding, Z. Li, Aeromagnetic data and geological structure of continental China: A review. Appl. Geophys. 13, 227–237 (2016).

3. S. Xiong et al., Aeromagnetic Data and Geological Structure of Continental China (Geological Publishing House, 2016).

4. C. Gsj, Data from "Magnetic anomaly map of east Asia 1: 4,000,000" (2024) Deposited 1 November 2024.

5. H. Zhang et al., Seismically imaged lithospheric delamination and its controls on the Mesozoic magmatic province in south China. Nat. Commun. 14, 2718 (2023).

Other supporting material for this manuscript:

Datasets S1 Crustal-scale analytical signal amplitude (ASA) model of East Asian (ASA model of East Asian.txt)
